# Aortic Dissection Presenting With a Seizure in a Young Woman: A Case Report

**DOI:** 10.7759/cureus.99256

**Published:** 2025-12-15

**Authors:** Rahma A Alibare, Rinsila R Hafthar, Latif Rahman

**Affiliations:** 1 Internal Medicine, University Hospitals of Leicester, Leicester, GBR; 2 Acute Medicine, Leicester Royal Infirmary, Leicester, GBR

**Keywords:** aortic dissection, aortic dissection (ad), atypical chest pain, chest pain, hyperten, neurological symptom, seizure activity, tearing, type a aortic dissection, undiagnosed hypertension

## Abstract

Aortic dissection is a rare but fatal emergency. It typically presents with sudden, severe chest pain or back pain, but can manifest atypically, complicating the diagnosis. We report a case of a 44-year-old woman who is a thalassemia carrier and presented with a seizure, without preceding chest or back pain. Computed tomography (CT) angiography revealed a penetrating atherosclerotic ulcer causing a dissection originating from the mid aortic arch, with retrograde extension into the proximal innominate artery and inferior extension down the descending thoracic and abdominal aorta, terminating at the infrarenal aorta.

## Introduction

Aortic dissection is a rare, fatal vascular event characterized by tearing of the intimal layer of the aortic wall, causing blood pooling into the space between intima and media, causing a false lumen [1]. It has a high mortality if left untreated [1]. The common presentation is sudden, severe chest pain, but there are unusual presentations that often lead to misdiagnosis [1]. 

The Stanford classification categorizes aortic dissections into two types based on whether the ascending or descending aorta is affected [1]. Type A involves the ascending aorta, regardless of where the primary intimal tear occurs, and is defined as a dissection that occurs before the brachiocephalic artery [1]. Type B originates beyond the left subclavian artery and affects only the descending aorta [1].

The incidence of aortic dissection is reported to be 5-30 cases per million people, with type A dissections twice as common as type B dissections [1]. The male-to-female ratio is 2:1, with women presenting later in life [2]. The mortality rate in type A dissection is 0.12% per hour during the first 48 hours, making this the most vulnerable time frame [3].

Age is a key risk factor for aortic dissection, most commonly affecting individuals between 40 and 70 years of age [2]. In 75% of cases, recurrence occurs between 50 and 65 years of age [2].

Although the classical presentation of aortic dissection is sudden, severe *tearing *chest pain, this is not always the case. It may also present with neurological deficits. In this case report, we describe a young woman who presented with a seizure, in whom a prompt general physical examination led to the diagnosis of aortic dissection.

## Case presentation

A 44-year-old female presented with a first-onset seizure at her workplace. The event was followed by a prolonged postictal phase. There were no associated symptoms, and no obvious trigger for the seizure was identified. Her past medical history was significant for thalassemia trait. Two weeks before this presentation, she had been reviewed in the same-day emergency care for high blood pressure. Ophthalmology assessment at that time revealed grade 4 hypertensive retinopathy. Antihypertensive therapy with amlodipine was initiated. She is a non-smoker and has no significant family history.

On arrival, she was tachycardic with normal blood pressure. Her observations are summarized in Table 1. 

**Table 1:** Observation on arrival. mmHg, millimeters of mercury

Type	Value	Reference range
Blood pressure	106/67 mmHg	90-120 mmHg systolic; 60-80 mmHg diastolic
Respiratory rate	16 breaths per minute	12-20 breaths per minute
Heart rate	126 beats per minute	60-100 beats per minute
Oxygen saturation	96% on air	95%-100% on room air

During her stay in the emergency department, she complained of left scapular pain but denied chest pain, palpitations, or dizziness.

On examination, she was alert but appeared distractible. She was tender in the left scapular region and had reduced air entry in the left lung field.

**Figure 1:**
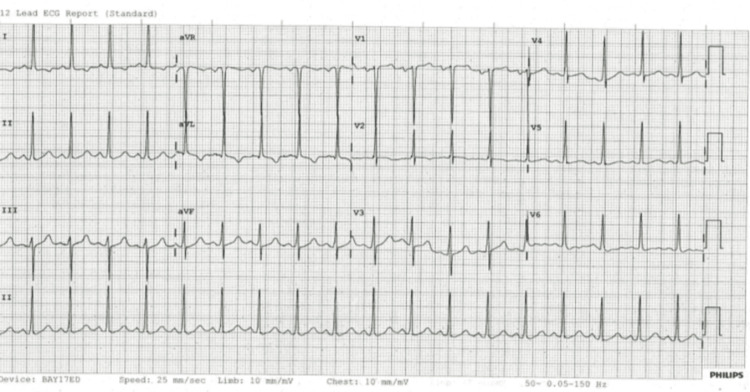
ECG showing sinus tachycardia with T-wave inversion in leads I and aVL. ECG, electrocardiogram

The ECG showed sinus tachycardia with T-wave inversion in leads I and aVL (Figure 1).

Her blood on arrival showed raised inflammatory markers, low hemoglobin, raised urea, raised creatinine, and low potassium. The results are summarized in Table 2.

**Table 2:** Laboratory investigation findings. WBC, white blood cells; Hb, hemoglobin; Hct, hematocrit; Plt, platelet count; Na, sodium; K, potassium; Mg, magnesium; PO4, phosphate

Laboratory investigations	Results	Reference range	Unit
WBC	30.9	4.0-11.0	x10^9 g/L
Hb	78	115-165	g/L
Plt	692	140-400	x10^9 g/L
Hct	0.251	0.370-0.470	L/L
Neutrophils	27.07	1.50-7.50	x10^9 g/L
Na	134	133-146	mmol/L
K	2.5	3.5-5.3	mmol/L
Mg	0.89	0.70-1.00	mmol/L
Adjusted calcium	2.37	2.12-2.51	mmol/L
PO4	2.18	0.8-1.5	mmol/L
Creatinine	168	60-120	umol/L
Urea	8.8	2.5-7.8	mmol/L

The computed tomography (CT) scan of the head showed no intracranial abnormalities.

**Figure 2:**
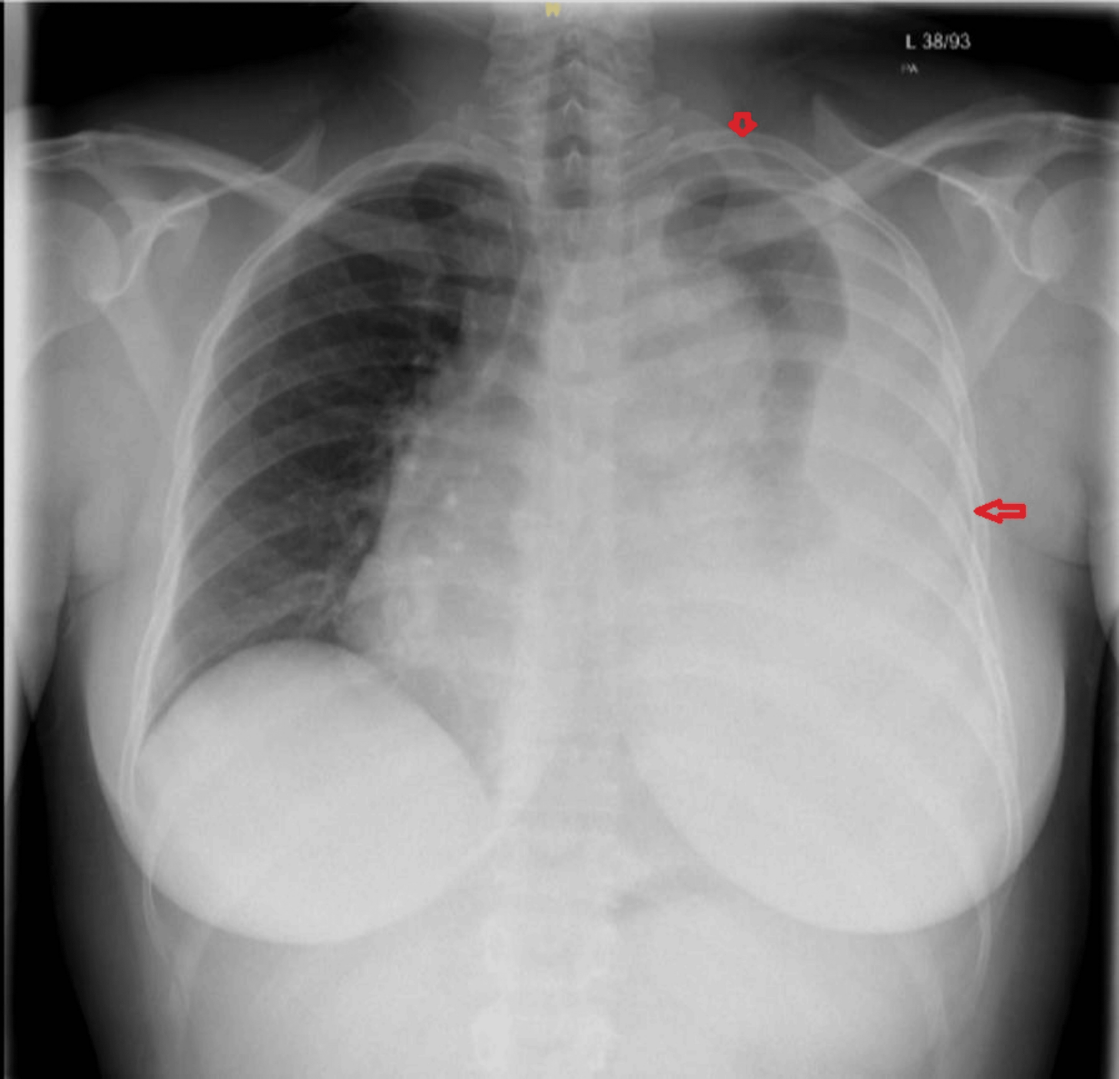
Chest X-ray showing a large left pleural effusion with partial collapse of the left lung, accompanied by a slight mediastinal shift to the right. The top arrow points to the partial collapse of the left lung, and the left horizontal arrow points to the large left pleural effusion.

**Figure 3:**
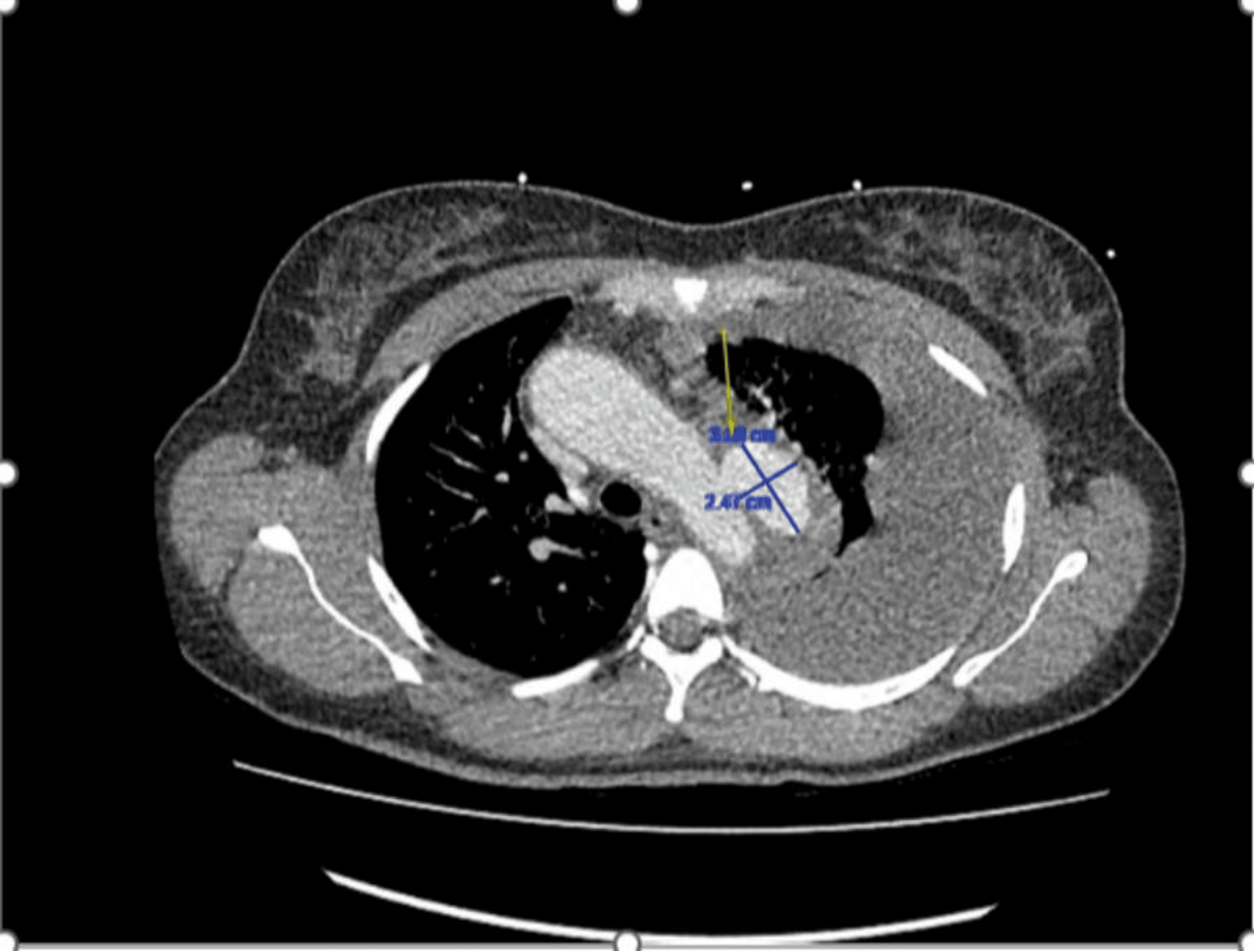
CT thorax showing a proximal descending aortic pseudoaneurysm with a large left hemothorax (yellow arrow), consistent with a contained rupture.

Chest X-ray revealed a large left pleural effusion with partial collapse of the left lung, accompanied by a slight mediastinal shift to the right (Figure 2). The pleural effusion was confirmed as a hemothorax resulting from a ruptured penetrating atherosclerotic ulcer originating from the mid aortic arch (Figure 3).

CT thorax showed a proximal descending aortic pseudoaneurysm with a large left hemothorax, consistent with a contained rupture, possibly related to a penetrating atherosclerotic ulcer and with no history of prior trauma. This was subsequently followed by a CT angiogram.

**Figure 4:**
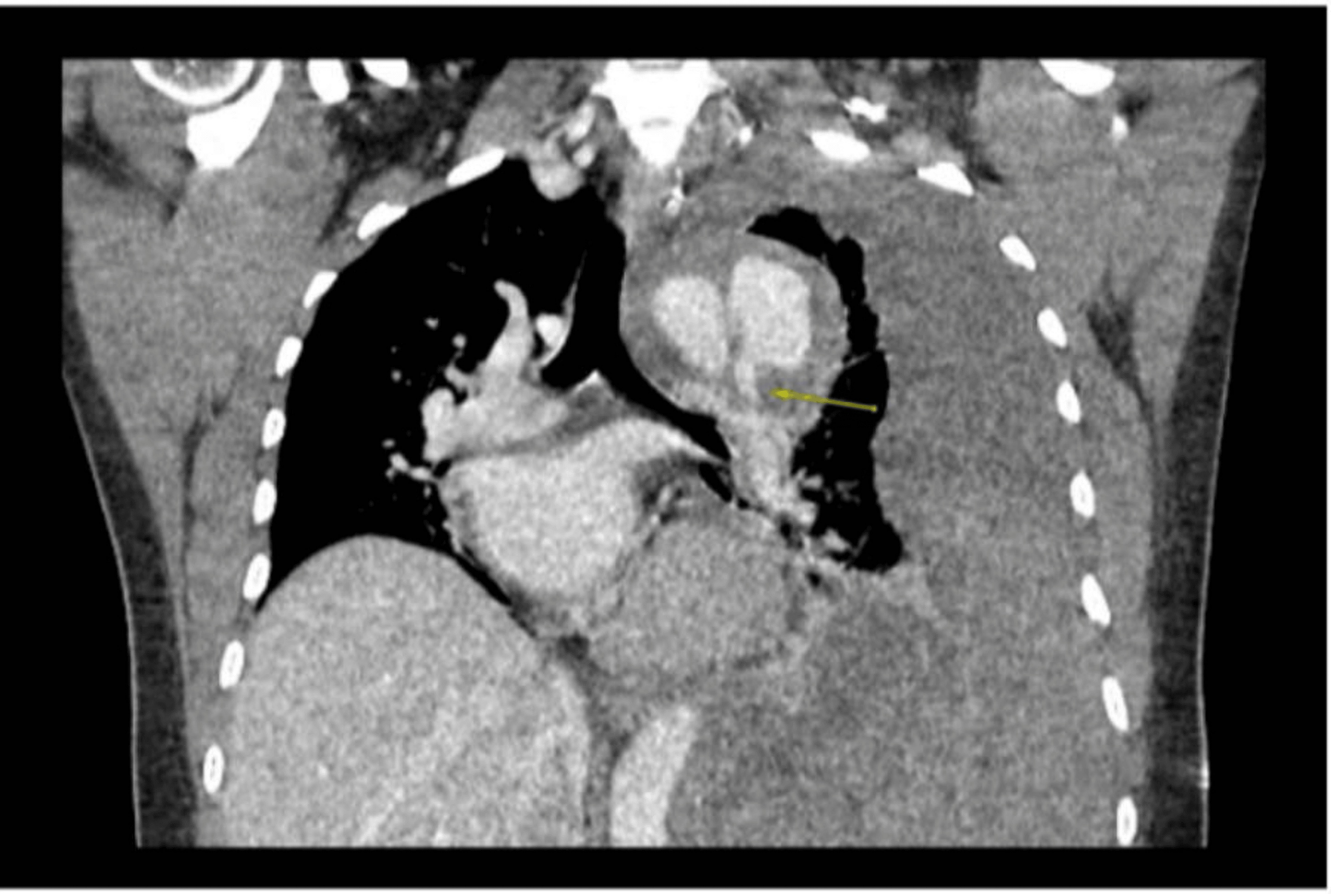
CT thorax showing a proximal descending aortic pseudoaneurysm with a large left hemothorax (arrow), consistent with a contained rupture, possibly related to a penetrating atherosclerotic ulcer and with no history of prior trauma.

CT angiogram revealed a penetrating atherosclerotic ulcer causing a dissection originating from the mid aortic arch, with retrograde extension into the proximal innominate artery and inferior extension down the descending thoracic and abdominal aorta, terminating at the infrarenal aorta (Figures 3-4). This qualifies as a Stanford type A aortic dissection [1]. 

She was commenced on empirical broad-spectrum antibiotics and covered with intravenous antivirals to address the possibility of meningitis and/or encephalitis. Concurrently, intravenous potassium chloride was administered to correct her profound hypokalemia, and fluid therapy was initiated to support her acute kidney injury. Following confirmation of the CT thorax, beta-blocker infusion was promptly started to reduce aortic shear stress.

Her care was subsequently taken over by the cardiothoracic surgery team and vascular surgery team, who managed the ruptured thoracic penetrating ulcer and dissection with thoracic endovascular aortic repair (TEVAR) and left chest drain, in addition to left subclavian bypass. Postoperatively, she was unable to mobilize her lower limbs but regained function after. MRI later confirmed that she had subacute infarcts secondary to hypotension rather than embolic events. She subsequently had an outpatient follow-up for her hypertension and stroke.

## Discussion

The aortic wall has three layers: intima, media, and adventitia. Aortic dissection is a rare life-threatening vascular emergency characterized by the tearing of the intima of the aortic wall. This causes blood to flow through the space between the intima and media, forming a false lumen. This can rupture into the mediastinum, pleural, and pericardial spaces. 

A 27-year population-based longitudinal study conducted by Mészáros et al. included 106,500 individuals, comprising 66 hospitalized and 18 non-hospitalized consecutively observed patients [4]. The findings revealed that aortic dissection was the initial clinical impression in only 13 of 84 patients (15%), indicating that 85% of patients did not receive prompt and appropriate medical treatment [4].

As aortic dissection commonly presents with chest pain, it can be misdiagnosed as acute coronary syndrome, and treatment with antiplatelet therapy may worsen the condition, potentially leading to patient death [5].

The classic presentation of an aortic aneurysm is sudden, severe “tearing” chest pain, reported in approximately 83% of cases, typically radiating to the back [2]. Neurological involvement, such as stroke-like symptoms, paresthesia, or limb weakness, is due to hypovolemia, arrhythmia, or myocardial infarction, and accounts for 20% of presentations [1].

Our patient presented with a seizure as the initial presentation of aortic dissection. She had a normal CT scan. The suspicion was raised after she started complaining of pain around the scapula. The absent breath sounds on auscultation of the left side prompted a chest X-ray, which revealed a large left pleural effusion with partial collapse of the left lung, accompanied by a slight mediastinal shift to the right. Chest X-ray features suggestive of aortic dissection include a widened mediastinum (greater than 8 cm), an abnormal aortic contour, pleural effusion, or loss of the aortic knob [1]. Additional radiographic clues may include a left apical cap, deviation of the esophagus or trachea, depression of the left mainstem bronchus, loss of the paratracheal stripe, and the presence of pleural effusion [1]. However, up to 20% of patients may have a normal chest X-ray; therefore, a normal result does not exclude the diagnosis [1]. The ECG may show ST-T changes if the dissection extends to the coronary arteries; however, a normal ECG cannot rule out aortic dissection [1]. Given the concerning features, CTA was performed, confirming the diagnosis of aortic dissection. CTA is the most sensitive and specific imaging modality for diagnosing aortic dissection, providing detailed visualization of the aortic anatomy and the extent of the dissection [6].

One of the major modifiable risk factors, hypertension, accounts for 70% of the cases and 1.9% for connective tissue disorders [7]. This patient had undiagnosed hypertension, which made her prone to aortic dissection. This patient was a thalassemia carrier, and a few case reports and older small series describe unusual connective-tissue-like changes or aneurysmal dilatation of the aorta in individual thalassemia patients (mostly intermedia/major), suggesting that rare, patient-specific mechanisms may exist [8]. However, case reports cannot establish population-level risk.

Although the hallmark presentation of aortic dissection is chest pain, neurological presentations such as stroke-like symptoms, limb weakness, or paresthesia are noted in 20% of cases [1]. A comparative study on neurological symptoms in Type A aortic dissections conducted by Gaul et al. involving 102 patients found that only two-thirds of them reported chest pain, while 29% presented initially with neurological deficits. The neurological manifestations were attributed to ischemic stroke (16%), spinal cord ischemia (1%), ischemic neuropathy (11%), and hypoxic encephalopathy (2%). Other common symptoms included syncope (6%) and seizures (3%) [9]. 

Zheng et al. reported a case of a 57-year-old woman who presented with loss of consciousness and a tonic-clonic seizure, followed by a manic episode. On examination, her blood pressure measured 145/90 mmHg in the left upper arm and 185/113 mmHg in the right upper arm. CT angiography (CTA) of the thoracic aorta revealed an aortic dissection originating from the lower portion of the ascending aorta and extending into the right common carotid artery, brachiocephalic artery, left common carotid artery, left subclavian artery, and descending aorta, with the false lumen opacified by contrast [10]. In this case, the patient had uncontrolled blood pressure, and an MRI of the head revealed high-signal ischemic lesions, indicating long-standing, uncontrolled hypertension [10].

Tannouri et al. presented a case of an extensive aortic dissection in a young male patient with atypical symptoms. The patient arrived at the emergency department with sudden-onset, severe chest pain and a seizure. Bedside transthoracic echocardiography (TTE) revealed a moderate pericardial effusion, correlating with the widened mediastinum seen on chest radiograph. The diagnosis of aortic dissection was subsequently confirmed by an urgent CT angiogram of the thorax and abdomen [11]. This case highlights the importance of bedside point-of-care ultrasound (POCUS) in aiding diagnosis. The patient presented with both chest pain and a seizure.

Srivastava et al. reported a case of a 29-year-old man with a history of hypertension who presented with altered mental status and seizures. According to family members, he had complained of cough, chest pain, headache, and fever before presentation. Laboratory investigations revealed mildly elevated high-sensitivity troponin levels, while chest X-ray showed cardiomegaly without acute changes. CT of the head demonstrated no acute abnormalities, and both EEG and lumbar puncture results were unremarkable. CTA revealed a type A aortic dissection originating distal to the sino-tubular junction and extending to the inferior mesenteric artery, involving the brachiocephalic trunk, left common carotid artery with severe stenosis, and the left subclavian artery. The patient was transferred to another facility for urgent cardiothoracic surgical evaluation. This case highlights that chest pain in the context of uncontrolled hypertension preceding neurological symptoms should raise suspicion for aortic dissection, even in young patients [12]. In this case, the diagnosis of aortic dissection could easily have been missed because the patient was confused and unable to communicate his history of chest pain. As many patients with aortic dissection present with altered consciousness, aphasia, or amnesia, obtaining an accurate medical history and identifying typical pain symptoms may be challenging, leading to potential delays in diagnosis [13].

## Conclusions

Aortic dissection, though rare, is a critical condition. This case highlights the importance of thorough A-E assessment in young patients presenting atypically with neurological symptoms, particularly in the context of hypertension and hypertensive end-organ damage. Prompt clinical examination, timely investigations, and early recognition are pivotal to facilitate appropriate management. 
